# Ki 67 assessment in breast cancer in an Egyptian population: a comparative study between manual assessment on optical microscopy and digital quantitative assessment

**DOI:** 10.1186/s13000-018-0735-7

**Published:** 2018-08-28

**Authors:** Essam Ayad, Ahmed Soliman, Shady Elia Anis, Amira Ben Salem, Pengchao Hu, Youhong Dong

**Affiliations:** 1 Department of Pathology, Cairo University, Cairo, Egypt; 2 Department of Oncology, XiangYang No.1 People’s Hospital Affiliated to Hubei University of Medicine, Xiangyang, Hubei 441000 People’s Republic of China

**Keywords:** Breast, Cancer, Ki67, Manual, Automated, Assessment

## Abstract

**Background:**

Breast cancer is by far the most frequent cancer among women. The proliferative index, Ki-67, is more and more taken into consideration for treatment decisions. However, the reliability of the established Ki-67 scoring is limited. Digital pathology is currently suggested to be a potential solution to Ki 67 assessment problems.

**Methods:**

This is a retrospective and prospective study including 100 patients diagnosed with invasive breast cancer. Three senior pathologists have been asked to estimate the Ki-67 proliferative index for each of the 100 cases by examining the whole glass slides on optical microscope and providing a continuous score then a categorical score (‘high’ and ‘low’ Ki 67 index) using once 14%, once 20% as threshold indicative of high Ki67 status. Finally, a digital quantitative assessment of Ki67 was performed.

**Results:**

A high inter-observer agreement was found when using optical microscopy for Ki 67 assessment, with correlation coefficient (CC) estimated at 0.878 (*p* value < 0.01). The overall agreement between manual and automated evaluation of Ki 67 was only substantial (CC estimated at 0.745 (*p* value < 0.01)). When using categorical scores, the inter-observers concordance was substantial using both cutoff points with kappa value estimated at 0.796 ([0.696–0.925] while using 14% as a cut off point and at 0.766 ([0.672–0.938] while using 20% as a cutoff point (*p* value < 0). The inter-observers agreement was better while using 14% as cutoff point. Agreement between manual and automated assessment of Ki 67 indices using both cutoff points was only substantial (Kappa estimated at 0.623, *p* value < 0.01). In comparison to automated assessment of Ki 67 index, while using 14% as a cutoff point, the overall tendency of all observers was to overestimate the Ki 67 values but to underestimate the proliferation index while using 20% as a cutoff point.

**Conclusion:**

Automated assessment of Ki 67 value would appear to be comparable to visual Ki 67 assessment on optical microscopy. Such study would help define the role of digital pathology as a potential easy-to use tool for a robust and standardized fully automated Ki 67 scoring.

*And Published in the* ***Virchows Arch (2016) 469 (Suppl 1):S1–S346*** *:*

*E. Ayad; “Evaluation of Ki-67 Index in invasive breast cancer: Comparison between visual and automated digital assessment” Virchows Arch (2016) 469 (Suppl 1):S191. DOI 10.1007/s00428-016-1997-7* [1].

## Background

Breast cancer is the most common malignancy affecting women in both developed and developing countries. It represents about 25% of all new cancer cases diagnosed in women per year. Fifty three percent of the newly reported cases are in developing countries, which represent about 82% of the world population [2].

Currently ER, PR and HER2 are recognized as prognostic and predictive factors [3, 4]. Ki-67 expression is more and more taken into consideration and has become a key factor for treatment decisions [5, 6].

Since 2011, the Saint Gallen guidelines stated that Ki 67 assessment allows for the segregation of the two types of luminal tumors (A and B) taking into account the value of the proliferation index. The application of chemotherapy is commonly recommended for patients with a high Ki-67 value [7, 8].

The 2011 Saint Gallen Consensus Meeting defined tumors with a Ki67 index of 14% or less as tumors with “low proliferation”. This cut-off was established by comparison with PAM50 intrinsic multigene molecular test classification of breast cancers [7, 9]. Then, during the 2013 Saint Gallen Conference, the majority of panelists voted for a threshold of 20% as indicative of “high” Ki67 status on the basis of many studies concluding that 20% is a significant factor for OS (overal survival) in the Luminal B subtype [8, 10].

However, during the Saint Gallen consensus meeting in 2015, the minimum value of Ki 67 required for the definition of luminal B subtype was for the majority of the panel ranging between 20 and 29% as many studies showed that patients with tumor with Ki67 > 20% showed the poorest prognosis [11, 12].

Because of the persistant intra and inter observers and laboratories variabilities, the panel of experts proposed finally that each laboratory define and use a median Ki67 value providing the best intra-laboratory inter-observer agreement as the cut-off distinguishing different subgroups [12].

Due to this dilemma about the cut off levels for Ki-67 suggested to distinguish prognostic subgroups, as well as the lack of standardization concerning preanalytical, analytical and methods used for interpretation and assessment of the Ki-67 score, this marker has not been implemented for routine clinical use in many pathological centers [5]. However during the last Saint Gallen meeting in 2017, the majority of the panelists agreed that the distinction between luminal A and luminal B subtypes by immunohistochemistry (IHC) approximate multigene testing results and 80% agreed that these two categories should be used for therapy decisions [13]. So an agreement on standardized method for Ki 67 evaluation is mandatory for proper management of breast cancer cases.

Digital pathology is an emerging field that is becoming more commonplace in routine pathology practice [14, 15].

Currently, there is interest in automating the assessment of Ki-67 labeling index with possible benefits in handling increased workload, with improved accuracy and precision.

### Aim of the study

To present and validate an easy-to-use, standardized and accurate Ki-67 scoring method in breast cancer by comparing observer’s performance on assessment of Ki-67 index on optical microscopy, then, by comparing the concordance between the results of the visual manual method and those of the automated Ki-67 assessment.

## Methods

### Patient’s cohort

We analyzed 100 cases of invasive breast cancer collected for the study from the pathology laboratory of Cairo university hospital during a period of 2 months (June–July 2015). Histological sections were obtained from paraffin blocks of 100 specimens (62 surgical specimens and 38 cores biopsies). Patient’s age ranges between 26 and 88 years old. Patients diagnosed with only ductal or lobular carcinoma in situ were excluded from the study.

#### Immunohistochemistry for Ki 67

4 μm thick sections were cut from paraffin-blocks which contained formalin fixed tumor tissue. During the whole staining procedure the slides were treated with the fully automated Benchmark Staining System (Ventana Medical Systems) using the primary antibody (rabbit monoclonal anti Ki − 67 human clone 30–09 Ventana Medical System). Then all the Ki 67 stained slides were scanned by iScan device [Produced by BioImagene (New Roche-Ventana)] present in the Digital Pathology Unit, Faculty of Medicine, Cairo University.

### Study design

Three different pathologists were asked to estimate the Ki-67 proliferative index for each of the 100 cases by examining the whole glass slide using *optical microscope* and to provide Ki-67 results using:

- ***Continuous score***: a score in the range of 0 to 100 corresponding to the percentage of positive tumors cells.
- ***Categorical scores***: The results provided by different observers were then classified into 2 categories using 2 different cutoff points;
- *First*: Cutoff point = 14%: patients were considered to have a ‘High Ki 67’ status if the observer judged that 14% of cells or more were positive for Ki-67 expression and ‘low Ki 67’ status otherwise.
- *Second*: Cutoff point = 20%: patients were considered to have a ‘High Ki 67’ status if the observer judged that 20% of cells or more were positive for Ki-67 expression and ‘low Ki 67’ status otherwise.

Stained nuclei were considered positive regardless of the intensity of staining. A whole slide average score, including hot spot areas, was the method used to estimate the Ki 67 value in all cases.

All the pathologists performed Ki 67 assessment independently and were blinded to patient outcome as well as other observer’s results.

### Quantitative digital analysis of Ki67

The whole scanned slide was examined then; multiple snapshots were captured with a (× 40) objective covering almost the whole scanned slide (15–50 snapshots per case). Areas rich in tumor cells were preferably chosen. Areas showing necrosis or significant lymphocytic infiltrate were avoided to minimize false positive results. Finally, a digital quantitative analysis of Ki 67 proliferative index was performed corresponding to the digital quantitative assessment of the percentage of the positive tumour cells for Ki67 using **ImmunoRatio website** [including the algorithm needed for nuclear counting of both positive & negative nuclei]. The program made a primary step of pseudo-color image showing staining component followed by image analysis according to the scale selected for the analysis. For each case, the software was able to identify stained and unstained nuclei (regardless of the intensity of staining) and to provide a percentage of positive nuclei for each snapshot. Then the final value of Ki 67 index of the case was calculated taking the arithmetic mean of all Ki 67 index values for each image individually.

### Statistical analysis

Inter-observers agreement was analyzed for each case, using categorical scores (with the two different cutoffs) and continuous scores. A study of agreement between Ki 67 assessment results provided using optical microscopy in comparison to the digital quantitative assessment of Ki 67 was also done.

### Statistical tests used for data management

Data was tabulated and analyzed using the computer program SPSS (Statistical package for social science) version 16. In the statistical comparison between the different groups, the significance of difference was tested using one of the following tests:

1. Kappa (κ): The interobserver agreement for each pair of observers was estimated then a mean of the kappa values was calculated. Kappa was interpreted as following: 0.0–0.20: Slight agreement, 0.21–0.40: Fair agreement, 0.41–0.60: Moderate agreement, 0.61–0.80: Substantial agreement, 0.81–1.00: Almost perfect agreement [16].
2. Correlation coefficient (CC): The inter-observer agreement for each pair of observers was estimated then a mean of the CC values was calculated.

There is no universally accepted standard criteria for the CC, the following criteria, similar to the kappa coefficient were used here to aid interpretation: 0.00–0.20 was interpreted as “slight correlation”; 0.21–0.40 as “fair correlation”; 0.41–0.60 as “moderate correlation”; 0.61–0.80 as “substantial correlation”; and > 0.80 as “almost perfect correlation” [17].

A ‘*p’* value < 0.05 was considered statistically significant (*) while > 0.05 statistically insignificant *‘p’* value < 0.01 was considered highly significant (**) in all analyses.

## Results

One hundred cases of invasive breast carcinoma were included in this study. The patients were 99 women and one man and ranged in age from 26 to 88 years old with a mean age of 55.46 years old.

The majority of cases were diagnosed as invasive duct carcinoma, with only one case of invasive lobular carcinoma. Invasive tumors were classified as grade 1 in 4% of cases, as grade 2 in 73% of cases and as grade 3 in 13% of cases. Regarding the hormonal receptors and HER 2 profile, 79% of cases were ER and/or PR positives, 14% of cases were triple negatives and 7% of cases were only Her 2 positives.

### Inter-observer variability on optical microscopy using continuous scores (Fig. 1)

The correlation coefficient (CC) runs to determine the relationship between Ki 67 assessment performed by the 3 observers showed an almost perfect agreement (CC: 0.878, *p* value < 0.01).

**Fig. 1:**
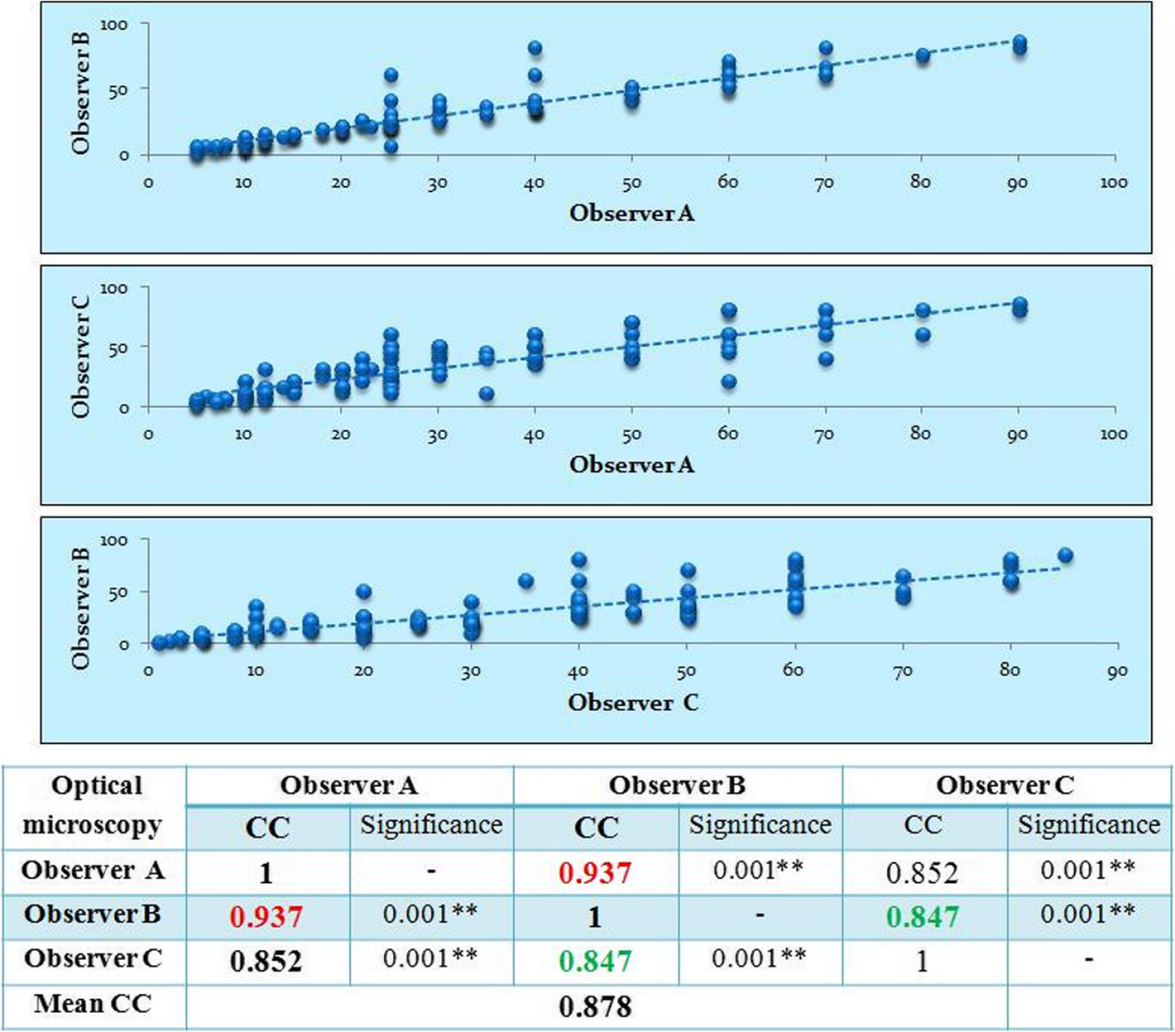
Inter-observer variability on optical microscopy using continuous scores

The main groups with highest variable Ki 67 index values were the groups with Ki 67 value varying between 11 and 35%, and for some pairs of observers the most discordant values were within the group with Ki 67 index between 15 and 25%.

### Inter-observer variability on optical microscopy using categorical scores (Fig. 2)

*While using 14% as a cutoff indicative of ‘High Ki 67’ status:* 67% to71% of cases were classified as having high Ki 67 index and 21% to 26% were classified as having low Ki 67 index at least by 2 observers. However 3% to 12% of cases were variably classified by the observers. The Kappa coefficient used to evaluate the inter-observers variability showed a substantial agreement between the 3 observers (kappa: 0.796, *p* value< 0.01).

**Fig. 2:**
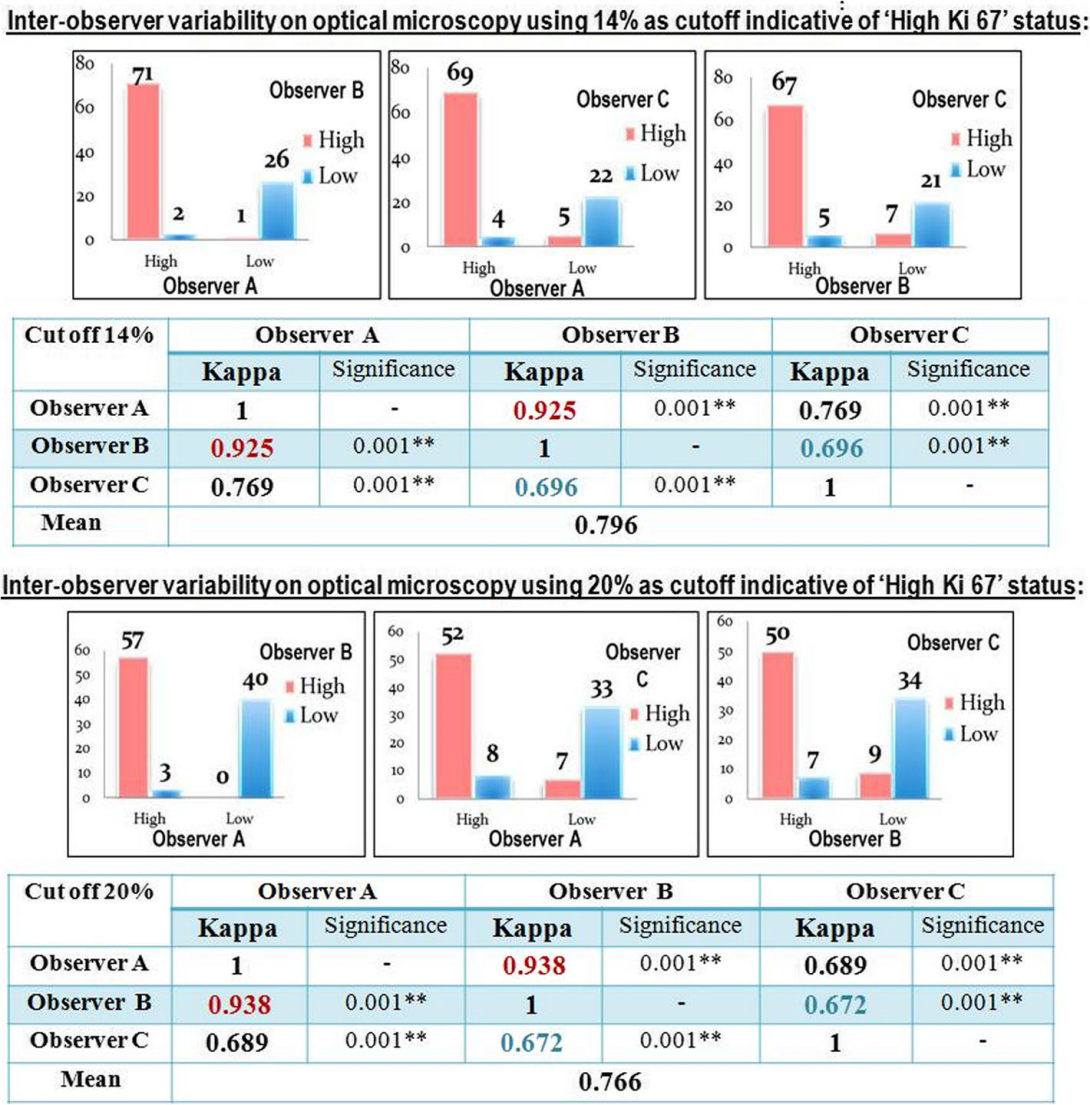
Inter-observer variability on optical microscopy using categorical scores

*While using 20% as a cutoff indicative of ‘High Ki 67’ status:* 50% to 57% of cases were classified as having high Ki 67 index and 33% to 40% were classified as having low Ki 67 index at least by 2 observers. However 3% to 16% of cases were variably classified by the observers. The inter-observers agreement was substantial with kappa value, slightly lower than the kappa value found with the cut off 14%. (Kappa: 0.766, *p* value< 0.01).

### Comparison of Ki 67 assessment results on optical microscopy and automated quantitative analysis results using continuous scores (Fig. 3)

The overall agreement between the manual and automated evaluation of Ki 67 was substantial with CC estimated at 0.745 (*p* value < 0.01).

**Fig. 3:**
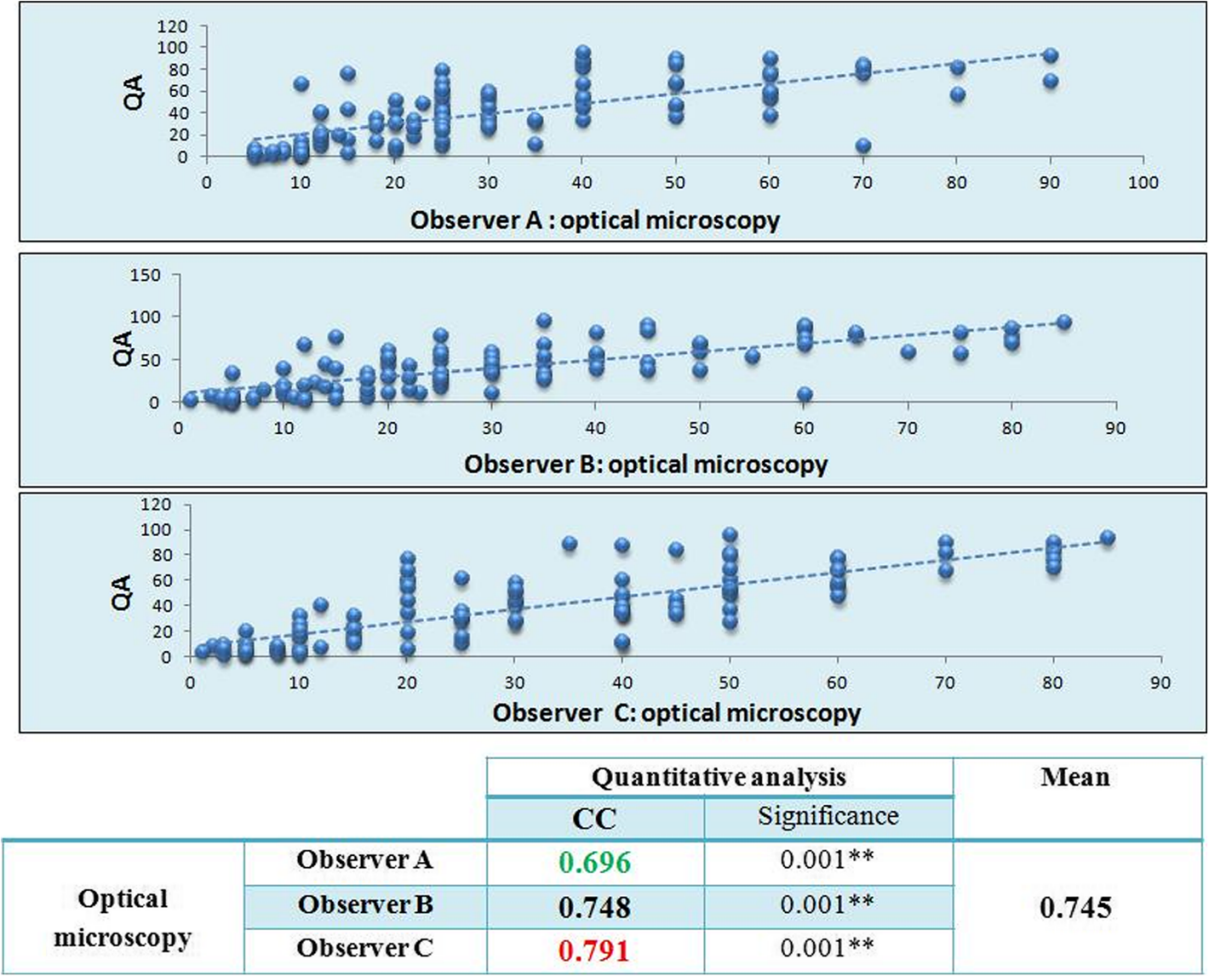
Correlation between manual assessment of Ki67 on optical microscopy and automated assessment results using continuous scores

### Comparison of Ki 67 assessment results on optical microscopy and automated quantitative analysis results using categorical scores

*While using 14% as a cutoff point*, 88.2% to 90.8% of the cases classified as having high Ki 67 index by quantitative analysis were classified as such by the 3 observers on optical microscopy and 75% to 79.2% of the cases classified as having low Ki 67 index by quantitative analysis were classified as such by the 3 observers on optical microscopy.

However, in 20.8% to 25% of cases the Ki 67 index was overestimated by the observers in comparison to automated assessment results and in 9.2% to 11.8% of cases the Ki 67 value was underestimated.

So, while using 14% as a cutoff point, the overall tendency was to overestimate the Ki 67 index using manual Ki 67 assessment in comparison to the automated method.

The overall agreement between manual and automated assessment of Ki 67 indices was only substantial (Kappa: 0.623, *p* < 0.01).

*While using 20% as a cutoff point*, 76.1% to 80.9% of cases classified as having high Ki 67 index by quantitative analysis were classified as such by the 3 observers on optical microscopy and 84.4% to 87.5% of cases classified as having low Ki 67 index by quantitative analysis were classified as such by the 3 observers on optical microscopy. However, in 12.5% to 15.6% of cases the Ki 67 index was overestimated by 3 observers in comparison to automated assessment results and in 19.1% to 23.9% of cases the Ki 67 value was underestimated.

So, while using 20% as a cutoff point, the overall tendency was to underestimate the Ki 67 index using optical microscopy in comparison to the automated method.

The overall agreement between visual assessment using optical microscopy and automated assessment of Ki 67 indices was also only substantial, but with Kappa value slightly lower than the kappa value found with the cut off 14 (Kappa: 0.602, *p* < 0.01).

## Discussion

Numerous studies have investigated the potential role of Ki67 as a prognostic marker as well as its role in predicting response to adjuvant and neo-adjuvant therapy. Although multiple meta-analyses showed that high Ki 67 index is associated with a higher risk of relapse and a worse survival in patients with early breast cancer [18, 19] and with a good response to neo-adjuvant chemotherapy [20], this marker is still not universally used in clinical routine. This is mainly due in one hand to the large inter-observer variability in assessment of the percentage of this marker, and in the other hand to the fact that clinical decision-making regarding treatment options in breast cancer often relies on the application of a Ki 67 cutoff to classify patients into “Ki67 high” or “Ki67 low” risk groups, however widely varying cutoff values (ranging from 0 to 28.6%) have been used to define the group with high Ki 67 [21, 22]. Also, several works reported that the lowest reproducibility of Ki67 results is mainly observed in the subset of cancers with intermediate proliferation activity (between 15 and 30%), the range in which most cutoffs are located for making clinical decisions [23–25], this further impede the clinical utility of Kin67 and make it difficult to compare Ki67 data across different studies.

Our study was designed to compare two different Ki 67 assessment modalities: visual estimation on optical microscopy and quantitative automated analysis. It also aimed to assess the reproducibility of both Ki 67 cutoffs proposed by the Saint Gallen Consensus Meetings in 2011 and 2013 to classify the tumors as having ‘high’ or ‘low’ Ki 67 index.

In contrast to many studies showing that inter-observer reproducibility of routine Ki-67 assessment in breast cancer on optical microscopy is poor to moderate especially in the grade 2 breast cancer group [26–28], the inter-observer agreement in our study was almost perfect agreement (CC: 0.878, *p* value < 0.01). However while comparing the results of Ki 67 assessment performed by different observers we found, as many studies showed [23–25], that the main group with highest variable Ki 67 index values was the group with Ki67 value varying between 11 and 35%, and for some pairs of observers the most discordant values were within the group with Ki 67 index between 15 and 25% (Fig. 1).

When we used the 2 cut off values (14% and 20%) to classify the cases as having “low Ki 67” or “high Ki 67” status, the inter-observer agreement on optical microscopy was only substantial using both cutoff points with kappa value when using 14% as cut off point, slightly higher than the kappa value found while using the cutoff 20% (Fig. 2).

A similar result was found by Varga Z et al. who showed higher inter-observer agreement while using the cutoff 14% in comparison to 20% but with lower kappa values than those found in our study with only slight to moderate agreement while using the 2 cutoff points (Kappa values 0.58 with cutoff 14% and 0.48 while using the cutoff 20%) [29].

According to these studies results, the inter-observer agreement was better using 14% as cutoff point although, according to other studies, 20% seems to be better reflecting the patient’s prognosis [30].

However, without standardization of the methodology, these cutoffs have limited value outside of the studies from which they were derived and the centers that performed them.

That is why researches have been conducted in order to develop other methods to ameliorate the inter-observer agreement and allow the reliable use of Ki 67 assessment as an additive factor for proper and consistent therapeutic decision.

Digital pathology is now a new approach used in many tasks [31, 32]. Many studies are now proposing the automated digital image analysis (DIA) as a potential efficient method of Ki67 index assessment, with benefits of increased precision and accuracy in comparison with visual evaluation or manual counting especially that it is tedious and labor intensive to count at least 1000 tumor cells, which has often been recommended for proper evaluation of Ki67 index [4].

A high correlation between manual and automated Ki 67 assessment have been showed by many studies which concluded that visual assessment and DIA both could be used for Ki67 assessment in clinical practice [33–35].

However, in our study, the overall agreement between the manual and automated evaluation of Ki 67 was only substantial with CC estimated at 0.745 (*p* value < 0.01) meaning that the correlation between manual and automated assessment methods is not always perfect.

So why to choose the automated Ki 67 index assessment?

In fact, recent studies showed that automated assessment of Ki 67 correlates better with clinical and pathological characteristics of breast cancer as well as the prognostic factors [36]. Gudlaugsson et al. concluded that Ki67 index assessment by DIA, but not subjective counts, was reproducible and prognostically strong [37].

Also, Stålhammar G et al. showed that all automated Ki 67 assessment methods are far better than the most meticulous manual assessment in terms of sensitivity and specificity, especially for the most diagnostic controversial subtype, the Luminal B subtype. Moreover, the level of agreement between the automated Ki 67 assessment results and the PAM50 gene expression assays was higher than that between the latter and the manual methods [38].

In our study, the overall agreement between manual and automated assessment of Ki 67 indices using 14% as well as 20% as cutoff points was only substantial.

The best kappa values reflecting the best consistency with quantitative analysis results were found while using 14% as a cutoff point. So for our laboratory, 14% seems to be a better reproducible cutoff point, with better inter-observer and inter-modalities agreement.

**Fig. 4:**
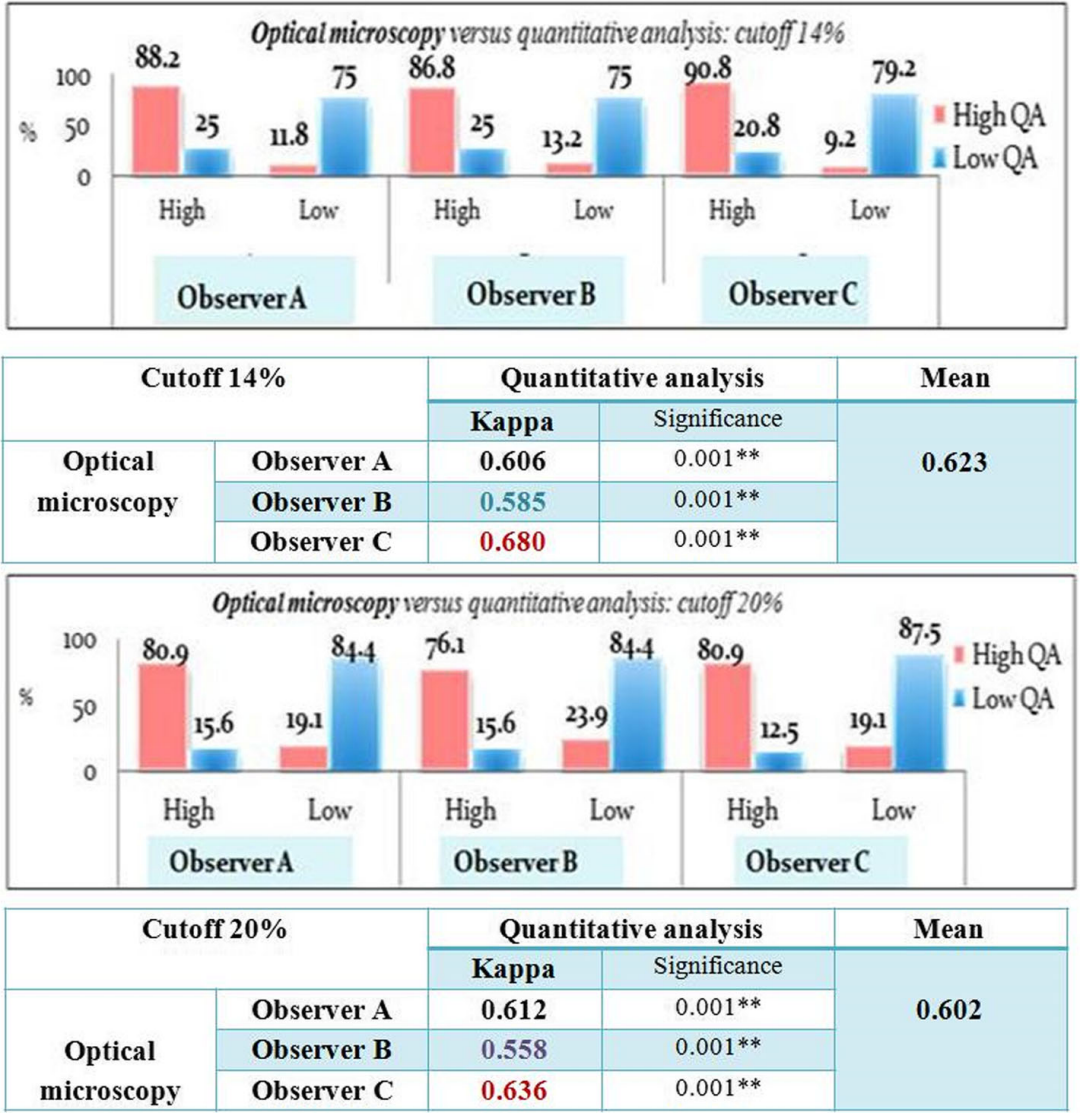
Correlation between manual assessment of Ki67 on optical microscopy and automated assessment results using categorical scores

However, In comparison to automated assessment of Ki 67 index, the overall tendency of all observers was to overestimate the Ki 67 values while using 14% as a cutoff point but to underestimate the Ki 67 values while using the cutoff point 20%. That means that the results of Ki 67 assessment for the group with Ki 67 indices varying between14 and 20%, the group in which most controversial cutoffs are located for making clinical decisions, were highly discordant between the 3 observers and quantitative analysis (Fig. 4).

Zhong F et al. study demonstrated similar results. For cases with high Ki 67 index (> 30%), DIA and visual assessment results were highly concordant. However, in cases showing intermediate Ki 67 index (11–30%), the agreement between both methods was only substantial to perfect [34].

By summarizing all these data, it seems that the computer-assisted quantitative analysis can improve the accuracy and inter-observer reproducibility of Ki 67 assessment and be a potential easy-to use tool for standardized fully automated Ki 67 scoring replacing the widely criticized current manual evaluation. This could prevent a wide proportion of patients from either receiving potentially harmful treatment such as cytotoxic chemotherapy without benefit or from being excluded from the beneficial treatment that a better diagnostic method would indicate.

However, DIA has some disadvantages. Some studies showed that automated assessment methods are less accurate than the visual ones in tumor cells identification, especially in tumors rich in lymphocytes, where some Ki67-positive lymphocytes may be identified as tumor cells. This will lead to Ki67 index value overestimation [34].

To overcome this problem, some authors proposed a method with semi-automated evaluation of Ki 67 index which allows for the determination of the exact proliferation index value by marking the immunostained tumor cells and the negative tumor cells manually then the cells are automatically counted and the ratio between immunomarked and negative cells gives the Ki 67 value [36, 39].

In our study, while choosing the areas to capture snapshots for automated quantitative analysis, we tried to avoid areas with significant lymphocytic infiltration to minimize the risk of over or underestimation of Ki 67 index.

*It should be mentioned that the findings of this study might not be generalized due to the large KI67 inter-laboratory variability.*

Newly developed DIA softwares tend also to overcome this problem by tissue classification using virtual double staining. For example, the same section will be stained for both cytokeratin and Ki67 markers; tumor cells are recognized by positive cytokeratin expression, and only cells that co-express both markers are automatically counted as positive tumor cells excluding any positive lymphocytes [38].

## Conclusion

Manual methods of Ki 67 assessment using optical microscopy lack perfect accuracy especially in cases with Ki 67 index ranging between 10 and 35% leading to improper distinction between Luminal A and B subtypes of breast cancer.

Further studies providing better techniques improving the accuracy of the automated Ki 67 assessment, especially identifying and detecting the tumor cells only, as well as trying to reduce the cost of this technique and make it more available, could help to consider automated assessment of Ki 67 as the most accurate and standard method and then could allow the universal agreement on a standard cut off that better distinguish the prognostic subgroups and concord with the molecular classification.

## Abbreviations

CC: Correlation coefficient
DIA: Digital image analysis
ER: Estrogen Receptor
HER2: Human epidermal growth factor receptor 2
IHC: Immunohistochemistry
PR: Progesteron Receptor
